# Diverse processing underlying frequency integration in midbrain neurons of barn owls

**DOI:** 10.1371/journal.pcbi.1009569

**Published:** 2021-11-11

**Authors:** Julia C. Gorman, Oliver L. Tufte, Anna V. R. Miller, William M. DeBello, José L. Peña, Brian J. Fischer

**Affiliations:** 1 Department of Mathematics, Seattle University, Seattle, Washington, United States of America; 2 Center for Neuroscience, University of California - Davis, Davis, California, United States of America; 3 Dominick P Purpura Department of Neuroscience, Albert Einstein College of Medicine, New York, New York, United States of America; National Research Council, ITALY

## Abstract

Emergent response properties of sensory neurons depend on circuit connectivity and somatodendritic processing. Neurons of the barn owl’s external nucleus of the inferior colliculus (ICx) display emergence of spatial selectivity. These neurons use interaural time difference (ITD) as a cue for the horizontal direction of sound sources. ITD is detected by upstream brainstem neurons with narrow frequency tuning, resulting in spatially ambiguous responses. This spatial ambiguity is resolved by ICx neurons integrating inputs over frequency, a relevant processing in sound localization across species. Previous models have predicted that ICx neurons function as point neurons that linearly integrate inputs across frequency. However, the complex dendritic trees and spines of ICx neurons raises the question of whether this prediction is accurate. Data from *in vivo* intracellular recordings of ICx neurons were used to address this question. Results revealed diverse frequency integration properties, where some ICx neurons showed responses consistent with the point neuron hypothesis and others with nonlinear dendritic integration. Modeling showed that varied connectivity patterns and forms of dendritic processing may underlie observed ICx neurons’ frequency integration processing. These results corroborate the ability of neurons with complex dendritic trees to implement diverse linear and nonlinear integration of synaptic inputs, of relevance for adaptive coding and learning, and supporting a fundamental mechanism in sound localization.

## Author summary

Neurons at higher stages of sensory pathways often display selectivity for properties of sensory stimuli that result from computations performed within the nervous system. These emergent response properties can be produced by patterns of neural connectivity and processing that occur within individual cells. Here we investigated whether neural connectivity and single-neuron computation may contribute to the emergence of spatial selectivity in auditory neurons in the barn owl’s midbrain. We used data from *in vivo* intracellular recordings to test the hypothesis from previous modeling work that these cells function as point neurons that perform a linear sum of their inputs in their subthreshold responses. Results indicate that while some neurons show responses consistent with the point neuron hypothesis, others match predictions of nonlinear integration, indicating a diversity of frequency integration properties across neurons. Modeling further showed that varied connectivity patterns and forms of single-neuron computation may underlie observed responses. These results demonstrate that neurons with complex morphologies may implement diverse integration of synaptic inputs, relevant for adaptive coding and learning.

## Introduction

How connectivity and neuronal properties jointly contribute to efficient and adaptable functions of neural networks remains a central question in neuroscience. It is known that similar response properties can be generated by circuits that differ in their connectivity or the biophysical properties of the component neurons [1–4] supporting degeneracy in neural coding [5]. However, how much each of these properties contribute to response selectivity and whether their interaction leads to degeneracy in building circuits with specific functions, remain open questions. Here we examined how circuit connectivity and the integrative properties of individual neurons contribute to the responses of space-specific neurons in the sound localization system of the barn owl.

The barn owl’s sound localization behavior is supported by a map of auditory space that emerges in the external nucleus of the inferior colliculus (ICx) [6,7]. A functional role of ICx neurons is to represent the direction of sound sources. The computations performed in the sound localization pathway that lead to a representation of auditory space in ICx have been extensively studied [8]. In particular, it is known that the spatial selectivity of ICx neurons in the horizontal direction is primarily due to their tuning to interaural time difference (ITD) [9–11]. ITD is initially computed in the owl’s sound localization pathway by neurons that are narrowly tuned to frequency [12]. Because of their narrow frequency tuning, the ITD-detecting neurons and their downstream targets have spatially ambiguous responses to ITD [12,13]. Spatial ambiguity is resolved in ICx neurons through the integration of information across frequency [14–18]. The mechanisms underlying frequency convergence in ICx are thought to be a linear subthreshold integration of ITD-dependent inputs, resembling a cross-correlation operation [15,18–23], followed by a spiking nonlinearity that further reduces the spatial ambiguity in ITD tuning [23,24]. While much of the physiological data from ICx neurons has been consistent with the linear-nonlinear processing sequence of point neuron models [19,23], recent anatomical data showing input clustering and large spines highlights the importance of testing whether nonlinear subthreshold processing also contributes to ICx responses [25,26]. While subthreshold multiplicative cue integration has been shown in these cells [27], this property could be achieved by a linear combination of inputs [23]. Additionally, nonlinear spiking responses of ICx neurons could be produced by a nonlinear conversion of membrane potentials to spikes [14,23,24,28]. In particular, it remains unknown whether frequency integration, a critical function for resolving spatial ambiguity across species [14,17,29], is underlied by linear or nonlinear processing of subthreshold responses of ICx neurons.

Two anatomically defined types of neurons have been observed in ICx [26]. Type I neurons have large toric spines that may integrate inputs across frequency channels, while type II have typical spines [26]. The large, thin shapes of the toric spines provide a possible substrate for compartmentalized, nonlinear dendritic processing in ICx [30]. Alternatively, these spines may facilitate linear integration of synaptic inputs [31]. However, physiological studies have not yet described nonlinear subthreshold processing across frequency in ICx.

**Fig 1:**
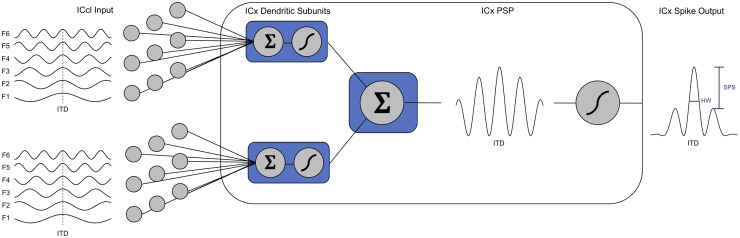
Two-layer model applied to ICx neurons. Left, the inputs to ICx neurons are model responses from lateral shell of the central nucleus of the inferior colliculus (ICcl) neurons, which are narrowly tuned to frequency and thus have approximately sinusoidal ITD tuning across frequencies. ICcl inputs tuned to same ITD (vertical dashed line) but different frequencies (F1-F6) are integrated in dendritic subunits of ICx neurons that apply a static nonlinearity to a weighted sum of the ICcl responses. Middle, the summation symbols (Σ) indicate linear integration operations within dendritic subunits and across subunit responses in the soma. The sigmoidal symbols (*∫*) represent nonlinear integration components determining a potential weighted sum (Σ-*∫*) in dendritic subunits and a spiking nonlinearity (*∫*) in the soma. The sum of subunit responses models the subthreshold postsynaptic membrane potential of the ICx neuron (ICx PSP). This subthreshold membrane potential is then passed through a spiking nonlinearity to model spiking responses of the ICx neurons. The connectivity between ICcl and ICx, and the forms of the subunit and spiking nonlinearities are varied to fit the data. Right, the measures of ITD tuning used to compare model and experimental responses are the half-width (HW) and side-peak suppression (SPS) of ITD tuning curves.

To test the hypothesis that ICx neurons perform nonlinear subthreshold integration of synaptic inputs across frequency, we analyzed *in vivo* intracellularly recorded responses of ICx neurons to tones and tone-combinations. Because frequency convergence in ICx involves the integration of inputs with narrower frequency tuning [13,14,32], manipulation of stimulus frequency is expected to determine the recruitment of independent synaptic inputs to these neurons, allowing to assess cellular response properties across different stimulus conditions. A two-layer model consisting of independent dendritic subunits whose outputs combine linearly to drive a spiking nonlinearity [33] was used to analyze subthreshold and spiking responses across sets of frequency combinations for each ICx neuron (Fig 1).

This two-layer model has been shown to accurately describe integration of synaptic inputs in biological neurons and in detailed compartmental models [33–40]. In addition, the two-layer model can describe clustering of synaptic inputs, which may be important for learning [41]. Physiological implications of clustering are still untested in these cells [42]. Analyzing the *in vivo* intracellular data with the two-layer model allowed us to test whether subthreshold frequency integration in ICx is linear or nonlinear and the cellular properties that could mediate this processing.

We found multiple types of neurons in ICx based on physiological frequency integration properties. Some neurons were best described by a two-layer model with nonlinear individual subunits, while other neurons were best described by a two-layer model with linear subunits. A circuit model, conducted to further test the nature of the inputs in the two-layer model, showed that many different network configurations are possible to produce the observed physiological responses. Additionally, a compartmental model considering specific biophysical mechanisms showed that there are multiple cellular properties that may produce the observed physiological responses. While many possible network configurations could predict responses, there was a systematic relationship between the degree of frequency integration over subunit inputs tuned to different frequencies and the shape of subunit nonlinearity: the broader the frequency integration, the more nonlinearly suppressive the subunit function must be to produce the observed ITD tuning. Even so, many forms of nonlinear responses were found to be possible in neurons with narrower frequency integration bandwidths, giving rise to observed ITD tuning. These results are consistent with the prediction that frequency integration occurs in diverse forms in ICx, allowing for a robust and flexible input processing in the midbrain auditory space map.

## Results

We used known properties of ITD and frequency tuning in ICx and its input region ICcl to infer the connectivity between ICcl and ICx, and the presence of nonlinear subthreshold frequency integration in ICx. We used a two-layer model of ICx neurons to investigate the role of nonlinear subthreshold processing in producing ICx responses. *In vivo* intracellular responses to sound were used to fit the spiking nonlinearity and test for nonlinearity in the first layer of the model. A circuit model was then used to determine the patterns of connectivity and types of subthreshold nonlinearity that were consistent with the data. Finally, a compartmental model was used to determine possible mechanisms for producing the subthreshold nonlinearities.

### Spiking nonlinearity in ICx

**Fig 2:**
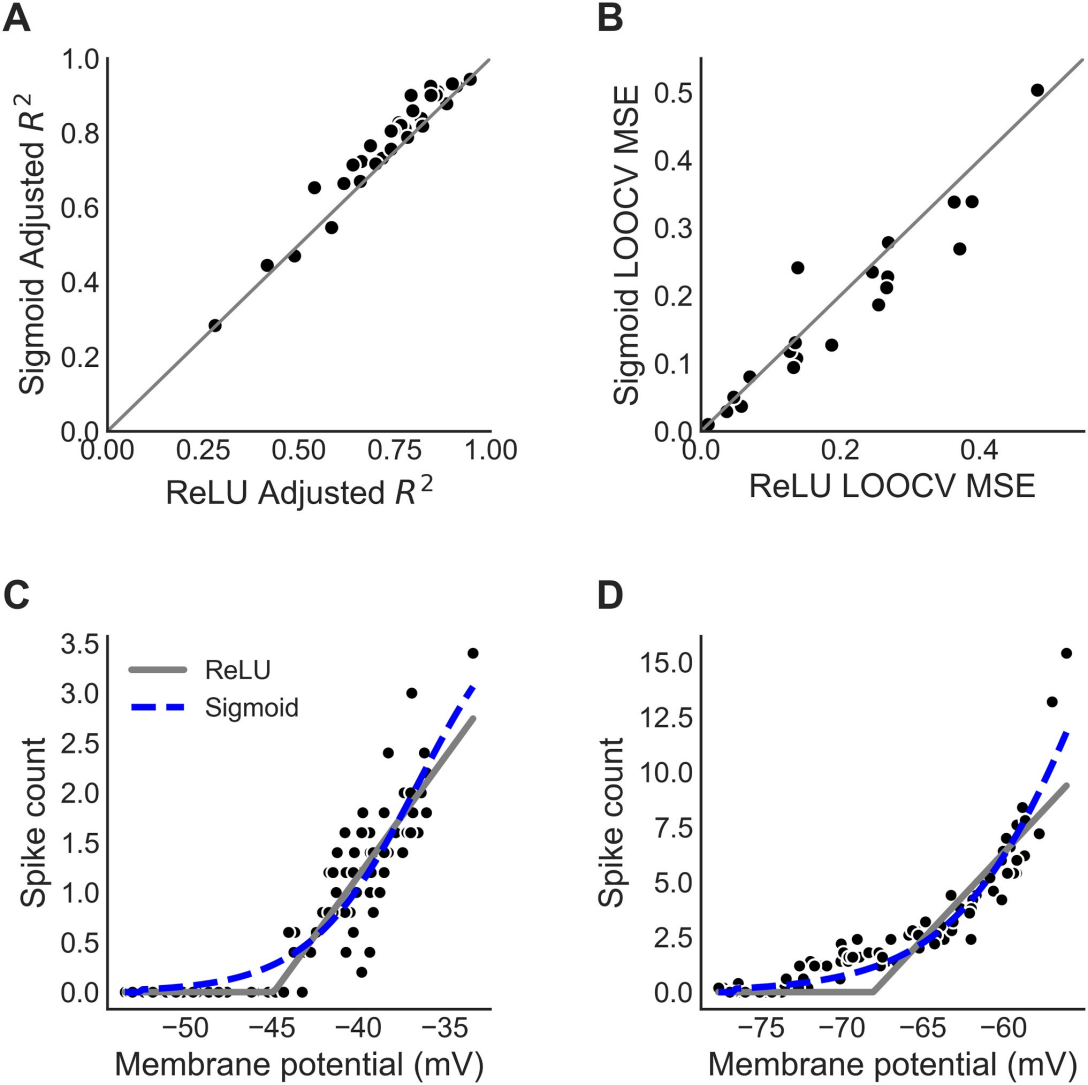
Spiking nonlinearity in ICx. (A,B) Comparison of the adjusted *R*^2^ (A) and leave-one-out cross validation (LOOCV) mean square error (MSE) (B) in the fits for the rectified-linear (ReLU) and sigmoid models for 32 ICx neurons. The solid line is the identity line. (C,D) Examples of spiking nonlinearities that are best fit by the ReLU model (C) and the sigmoid model (D). In C and D the ReLU model is shown in gray and sigmoid model is shown in blue.

The spiking nonlinearity in ICx was defined as the mapping from the trial-averaged median membrane potential to the trial-averaged spike count. The spiking nonlinearity was estimated using *in vivo* intracellularly recorded responses of ICx neurons (n = 37) to sounds where ITD, interaural level difference (ILD) and frequency were manipulated. Previous analysis showed that the spiking nonlinearity does not depend on these particular stimulus parameters [24], and therefore data were pooled over ITD, ILD, or frequency. We compared the fits obtained using rectified-linear and sigmoid nonlinearities (Fig 2). Five neurons had sparse spiking responses, which led to fits not being significant for any of the functions, and were excluded from further analysis. For the remaining 32 neurons, the spiking nonlinearity was typically well fit by both the rectified-linear and sigmoid nonlinearities, as assessed using adjusted-R^2^ (Fig 2A) and leave-one-out cross validation (LOOCV) mean square error (MSE) (Fig 2B) metrics (described in Materials and methods). However, the sigmoid nonlinearity performed better than the rectified-linear nonlinearity for a majority of neurons (23 of 32) due to the expansive, or concave up, form of the spiking nonlinearity near threshold values of the membrane potential that was observed for most neurons. While the differences in the adjusted-R^2^ and LOOCV MSE were small for most neurons, this finding of neurons with rectified-linear (Fig 2C) and sigmoid (Fig 2D) spiking nonlinearities has a functional significance, consistent with the presence of neurons with linear and nonlinear frequency integration properties in ICx [28]. Specifically, neurons with rectified-linear spiking nonlinearities where the input does not fall below threshold will have linear responses, while neurons with sigmoid spiking nonlinearities will have nonlinear responses. In particular, the sigmoid nonlinearity has an expansive portion near threshold that can contribute to multiplicative spiking responses [23,43,44] and further resolves spatial ambiguity in ITD tuning of ICx neurons [18,24,27].

### Testing nonlinear subthreshold frequency integration in ICx

We used *in vivo* intracellular measurements of ICx subthreshold postsynaptic potential (PSP) responses (n = 20 neurons) to tones and combinations of tones to test for nonlinear subthreshold frequency convergence in ICx. Two or three tonal stimuli were presented individually, then a stimulus consisting of a sum of the tones was presented [18]. We used the median of the intracellular trace over 50 ms of the stimulus duration starting 10 ms after onset as the response on each trial, where the median calculation limits the impact of spiking on response measurements [18,45]. Responses were then averaged over 3 to 5 trials. This allowed us to determine whether subthreshold frequency integration in ICx is nonlinear.

**Fig 3:**
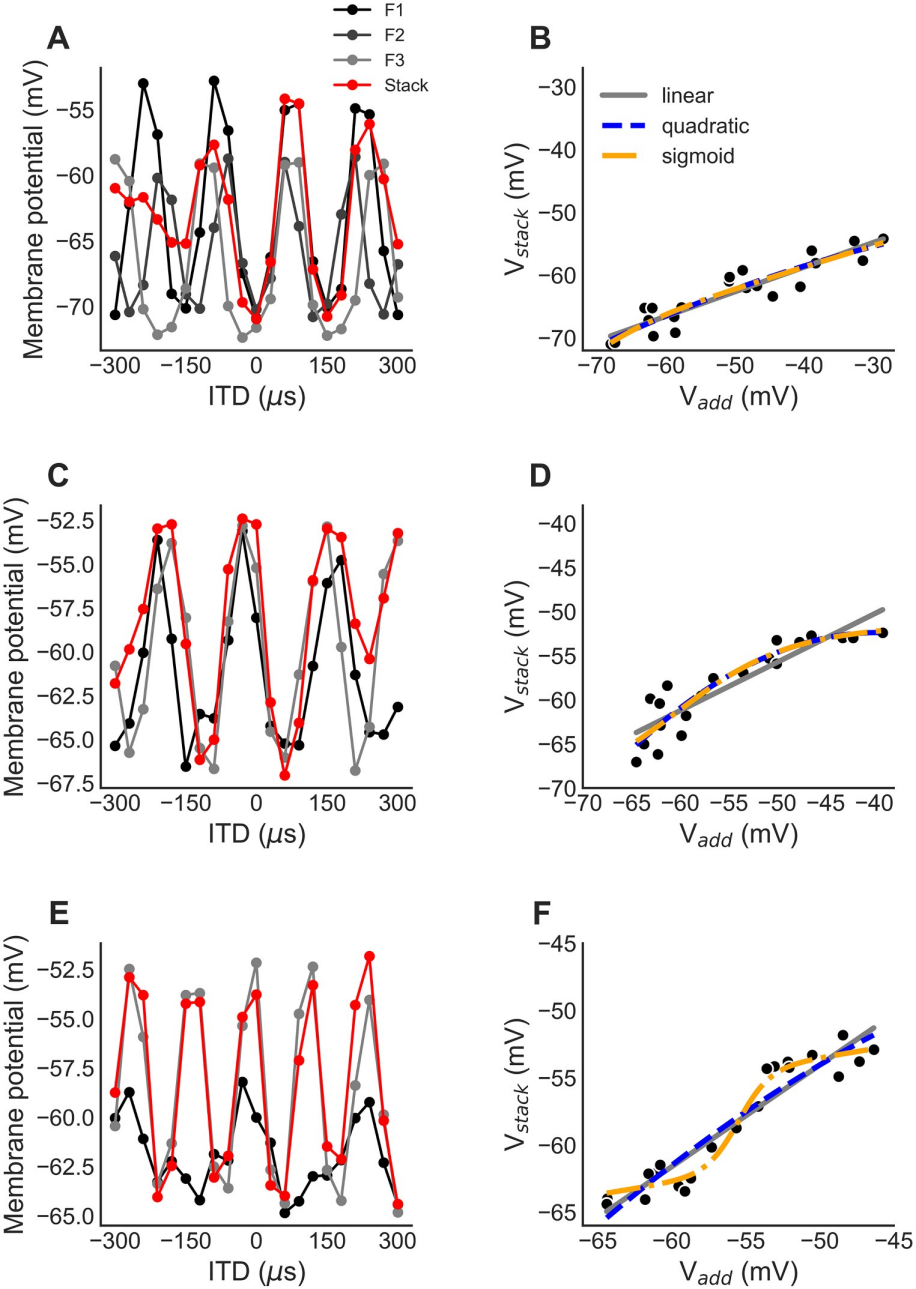
Subthreshold frequency integration in ICx. (A,B) Subthreshold PSP ITD tuning curves for tones (black and gray) and the tone combination (red) (A) for a neuron where the relationship between *V*_*add*_ and *V*_*stack*_ is linear (B). (C,D) As in (A, B) for a neuron where the relationship between *V*_*add*_ and *V*_*stack*_ is quadratic. (E,F) As in (A, B) for a neuron where the relationship between *V*_*add*_ and *V*_*stack*_ is sigmoidal. *V*_*add*_ is the sum of the PSP responses to individual tones relative to the minimum membrane potential recorded during stimulation and *V*_*stack*_ is the PSP response to the tone combination. The linear, quadratic, and sigmoidal best fits to the relationship between *V*_*add*_ and *V*_*stack*_ are shown in (B, D, and F) using solid gray, dashed blue, and dot-dashed yellow curves, respectively.

The PSP responses to the tone combination (*V*_*stack*_) for each ITD was compared to a linear combination of the PSP responses to the individual tones (*V*_*add*_) of the same ITD to test for nonlinear frequency integration in ICx subthreshold responses. The linear combination *V*_*add*_ is the sum of the responses to individual tones relative to the minimum membrane potential recorded during sound stimulation. We first observed that frequency integration was sublinear (*V*_*stack*_ < *V*_*add*_) for all neurons (Fig 3), consistent with the previous report [18]. This is expected here because the overall stimulus level was equal for the individual tones and the tone combinations. Thus, the components of the tone combination were presented at a lower sound level than when they were presented individually. We next determined whether the relationship between *V*_*add*_ and *V*_*stack*_ was described by a linear or a nonlinear function because a sublinear response could be produced by a linear process, such as an average, or a nonlinear process, such as saturation. Specifically, a linear fit between *V*_*add*_ and *V*_*stack*_ was compared to quadratic and sigmoidal fits to test for nonlinearity in frequency integration and best fit was assessed using adjusted R^2^ and LOOCV MSE metrics (Fig 3). We found cases of both linear (6 of 20 neurons) and nonlinear (14 of 20) frequency integration in subthreshold PSP responses. The majority of neurons showed nonlinear frequency integration, with the relationship between *V*_*add*_ and *V*_*stack*_ being best fit by either a quadratic model (3 neurons) or a sigmoidal model (6 neurons) according to both the adjusted R^2^ and LOOCV MSE metrics. In neurons where either the quadratic or sigmoidal model best fit the data (Fig 3D and 3F), there was a saturation of the PSP response to the tone combination (*V*_*stack*_) at high values of the sum of the PSP responses to the individual tones (*V*_*add*_). The sigmoidal model provided the best fit for neurons where the PSP response to the tone combination plateaued at both low and high values of the sum of the PSP responses to the individual tones (Fig 3F). The quadratic and sigmoidal responses observed are nonlinear responses to the tone combination and do not reflect merely a weaker linear response, such as an average. The linear model best fit the data in 6 of 20 neurons. This shows the presence of neurons in ICx that function as point neurons that linearly combine inputs across frequency. Consistent with the analysis of the spiking nonlinearity, this provides evidence of ICx neurons that perform linear frequency integration of PSPs, and ICx neurons that perform nonlinear frequency integration of PSPs.

Note that the presence of a sigmoidal relationship, for example, between *V*_*add*_ and *V*_*stack*_ does not mean that the subunit nonlinearity is determined by the sigmoidal function. A direct determination of the form of the subunit nonlinearity would require simultaneous measurement of the input and output of each subunit, which was not possible in these experiments. We can, however, use a model constrained by known ICcl and ICx responses to determine possible forms for subunit nonlinearities, addressed below.

### Possible forms of subunit nonlinearities

ITD tuning in ICx PSPs is determined by the bandwidth of frequency integration from ICcl and the form of subunit nonlinearities that are present in ICx neurons. We used a model to determine the combinations of frequency integration bandwidth and subunit nonlinearity shape that are consistent with ITD tuning in ICx. We used the ITD tuning half-width (HW) and side-peak suppression (SPS) of subthreshold PSPs measured *in vivo* for a sample of ICx neurons (n = 75) as constraints on the model [24].

**Fig 4:**
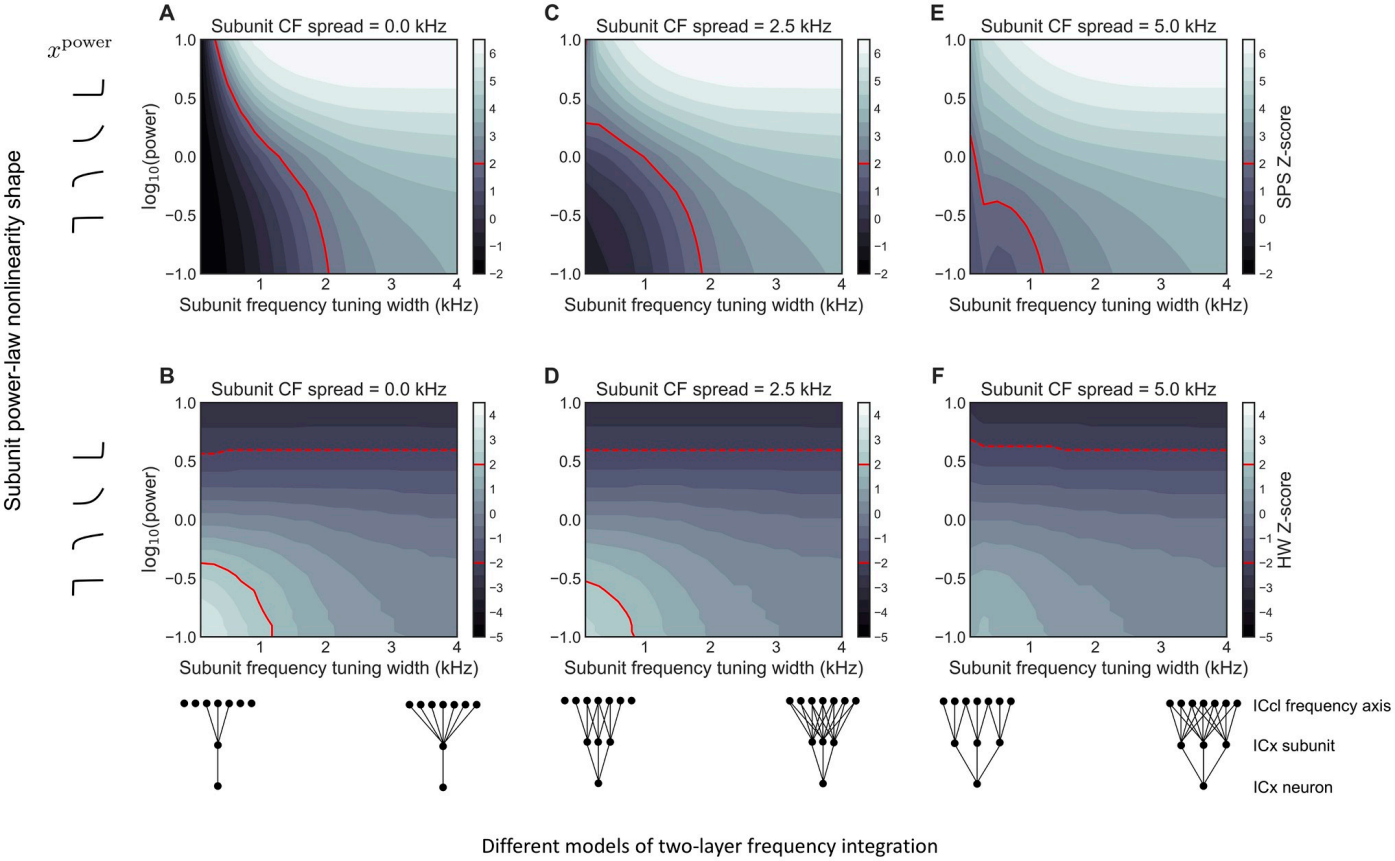
Possible ICx subunit forms. Z-scores of side-peak suppression (SPS) (A,C,E) and half-peak width (HW) (B,D,F) values for model ICx neurons, relative to experimentally measured values. The subunit frequency tuning width (x-axes) is the width of the Gaussian-shaped connection weights between the ICx subunit and the ICcl population. The log(power) (y-axes) is the log of the subunit power-law nonlinearity *x*^*power*^. The subunit power function is expansive when log(power) > 1, suppressive when log(power) < 1, but linear when log(power) = 1. The circuit diagrams at the bottom indicate that subunits have identical center frequencies in (A,B), small differences in center frequencies in (C,D), and larger differences in center frequencies in (E,F). Positive or negative z-score (gray color maps, defined to the right of each plot) indicate a model ITD curve where the SPS (top row) or HW (bottom row) was above or below, respectively, the average experimentally measured value. The red lines indicate z-scores of ±2. Values above the solid red line in A, C, E produce ITD tuning curves where SPS is larger than experimentally observed. SPS values did not have z-scores below -2, thus only one boundary line is present. Values above the dashed red line in B, D, F produce ITD tuning curves where HW is smaller than experimentally observed. Diagrams in the bottom row represent circuit configurations of subunits’ center frequency and frequency integration bandwidths across A-F plots.

In the two-layer model of ICx neurons, the frequency integration bandwidth is controlled by the amount of frequency integration within each subunit and the range of center frequencies across the subunits. For example, one mechanism to produce broad frequency tuning in an ICx neuron is to have broad frequency integration within a set of identical subunits (Fig 4A and 4B). This circuit configuration is illustrated by the circuit diagrams at the bottom of the left column. Here, all subunits have identical frequency integration bandwidths, but this bandwidth is varied between simulations. Another mechanism to produce broad frequency tuning in an ICx neuron is to have narrow frequency integration within each subunit but a wide range of frequency selectivity across subunits (Fig 4C–4F). The circuit diagrams at the bottom of the middle and right columns of Fig 4 illustrate the differences in spread between subunit center frequencies and the frequency integration bandwidth within each subunit that were varied to control the overall frequency tuning of the model neurons. Connectivity patterns between ICcl inputs and ICx subunits were varied to control the frequency integration bandwidth within subunits. In addition, the frequency tuning bandwidth of the ICx neuron was varied by increasing the range of subunit best frequencies.

Subunit nonlinearities were power functions, *g*_*i*_(*x*) = *x*^*p*^, that could be suppressive or expansive functions of their inputs, depending on the power *p*. The subunit nonlinearity is suppressive for *p* < 1 and expansive for *p* > 1. Suppressive and expansive subunits will decrease and increase, respectively, the SPS that is created by linear frequency integration within the subunits.

We found that many different configurations of subunit nonlinearity and frequency integration bandwidth produced ITD tuning consistent with experimental measurements (Fig 4). Fig 4 compares the SPS (top row) and HW (bottom row) in ITD tuning curves of model responses to the experimentally measured distributions of these values. The model’s SPS and HW predicted values for different subunit frequency integration bandwidths (horizontal axis) and subunit nonlinearity shapes (vertical axis) were assessed by computing their z-scores relative to the mean and standard deviation reported for ICx neurons [24]. A model was considered consistent with the experimental distribution if the z-scores for SPS and HW were within -2 (dashed red line) and +2 (solid red line), indicating that model responses are within two standard deviations of the experimental mean. Fig 4 shows that many different model configurations produce results that fall within these bounds.

In particular, neurons with suppressive, linear, and expansive subunit nonlinearities can have similar ITD tuning shape. While many different network configurations are possible, there was a consistent relationship between frequency integration bandwidth and the subunit nonlinearity shape. Specifically, for subunits with broad frequency integration, expansive subunit nonlinearities were not allowable because they led to ITD curves that were too narrow (Fig 4B, 4D and 4F) and where the SPS was too large (Fig 4A, 4C and 4E).

**Fig 5:**
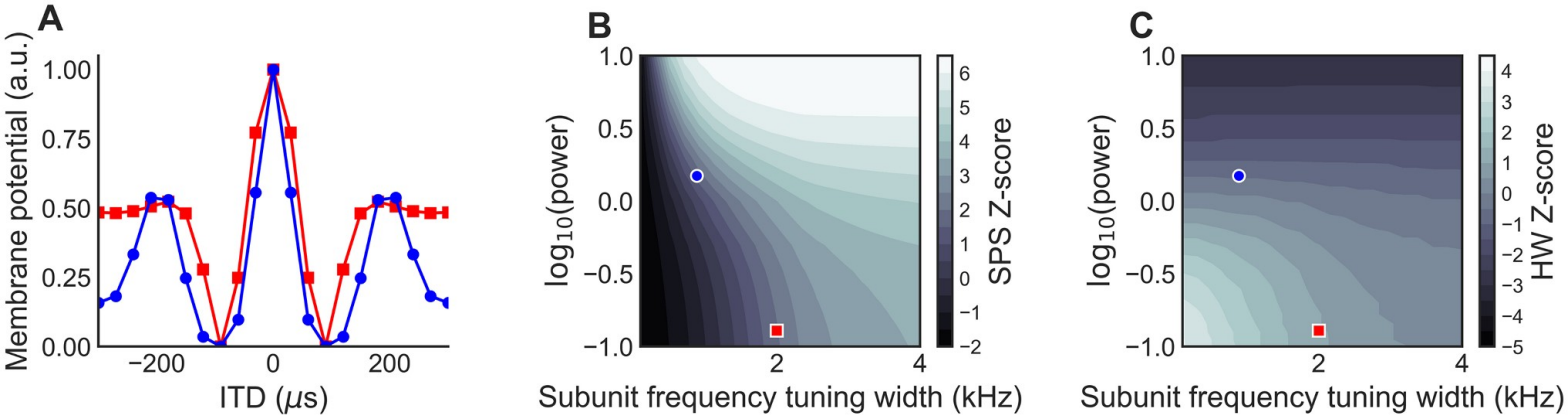
Similar responses from different models. (A) ITD tuning curves for two model neurons that have similar SPS and HW values. (B,C) Z-scores of SPS and HW as a function of subunit frequency tuning width as in Fig 4A and 4B. Blue and red dots on panels B and C, and corresponding colors of plots in A, indicate the two example neurons with different integration bandwidths and nonlinearity shapes.

We also found that model ITD tuning curves could look similar, even when they were created with very different parameters. The model ITD tuning curves in Fig 5A have similar SPS and HW values but are produced by neurons with different frequency integration bandwidths and subunit nonlinearity shapes (Fig 5B and 5C). This highlights the result that different anatomical and biophysical configurations of the ICcl-ICx circuit may produce functionally equivalent responses.

### Sigmoid as general subunit nonlinearity

**Fig 6:**
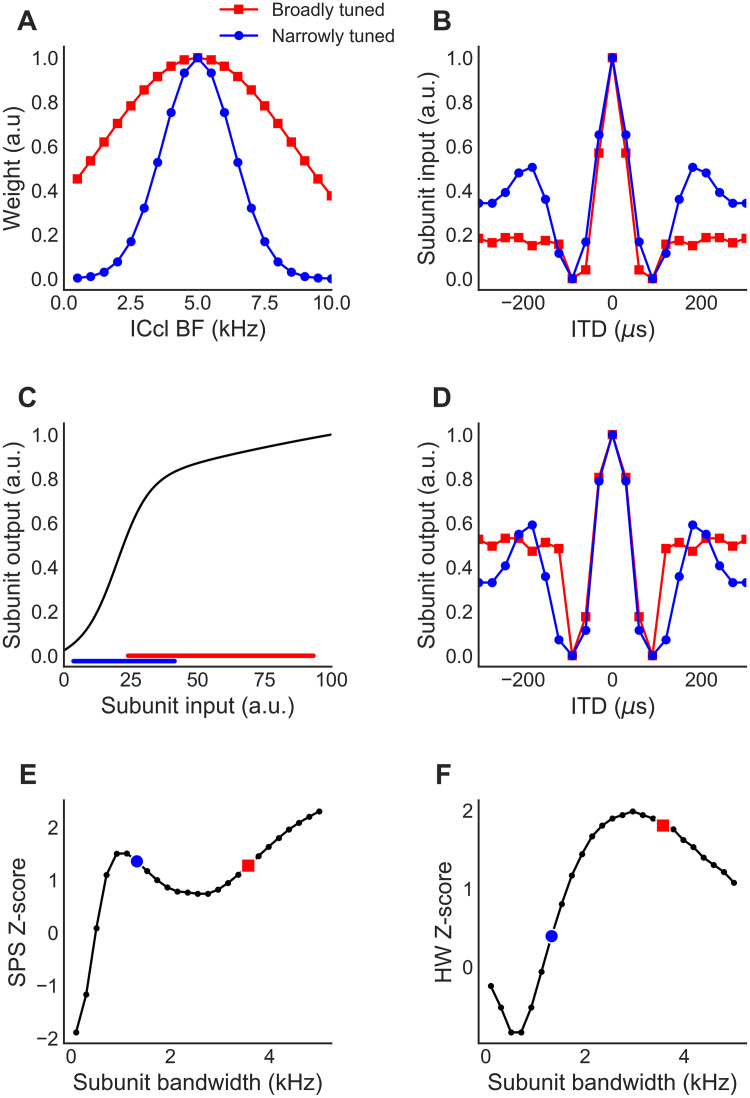
Sigmoidal subunit nonlinearity. (A) Connection weights between ICcl neurons and ICx subunits for two ICx-neuron subunits with different frequency tuning bandwidths, as a function of the best frequency (BF) of their ICcl inputs. The red curve is wider, reflecting the larger frequency tuning bandwidth. (B) ITD tuning of the linear input to the subunit. The side peaks are more greatly suppressed in the subunit with broader frequency tuning (red squares). (C) Sigmoidal subunit nonlinearity used in the model. The broadly tuned (red) and narrowly tuned (blue) inputs occupy different ranges of the sigmoidal function. (D) ITD tuning of the outputs of the subunits. (E,F) Z-scores of side-peak suppression (SPS) (E) and half-peak with (HW) (F) for subunit outputs with the sigmoidal subunit nonlinearity from (C) for different frequency tuning bandwidths. The examples shown in (A-D) are indicated with blue circles and red squares. In all panels, the CF spread of subunits was zero, as in the left column of Fig 4.

The previous analysis with a power-law subunit nonlinearity showed that the subunit nonlinearity cannot be expansive in the model when frequency integration is broad (Fig 4). We next tested whether a sigmoidal subunit nonlinearity with a fixed set of parameters would produce ITD tuning consistent with experimental observations, regardless of the bandwidth of frequency integration. The sigmoidal function is a candidate for a generally applicable model because it allows for both expansive and suppressive properties depending on the range of the curve on which the input exists. It allows stronger input that arises from broader frequency tuning to be suppressed, and weaker input from narrower frequency tuning to have an expansive nonlinearity, which is what the data suggest is necessary. We tested the two-layer ICx neuron having a sigmoidal subunit nonlinearity with fixed parameters and different frequency tunings (Fig 6). We tested conditions where the frequency integration bandwidth across ICcl inputs to the sigmoidal subunit was broad (Fig 6A red) and where it was narrower (Fig 6A blue). Correspondingly, the ITD tuning of the input to the sigmoidal subunit shows larger SPS when the frequency integration bandwidth is broader (Fig 6B) [15,18]. In addition to the differences in ITD tuning of the input in the two conditions, the magnitude of the input to the sigmoidal subunit is larger in the broadly tuned condition because inputs are integrated over a larger set of ICcl neurons (Fig 6C). Therefore, in the broadly tuned condition, the input occupies the suppressive part of the sigmoidal nonlinearity and the SPS of the ITD tuning in the output of the subunit is smaller than in the input. Conversely, in the narrowly tuned condition, the inputs fall on the expansive and linear parts of the sigmoidal nonlinearity and the SPS in the output of the subunit is larger than in the input. The sigmoidal model therefore produced subthreshold ITD tuning that was consistent with experimental observations over a wide range of frequency tuning bandwidths (Fig 6E and 6F). This suggests that a sigmoidal subunit nonlinearity can be a general-purpose functional unit for locally combining inputs across frequency in ICx neurons.

### Compartmental model

**Fig 7:**
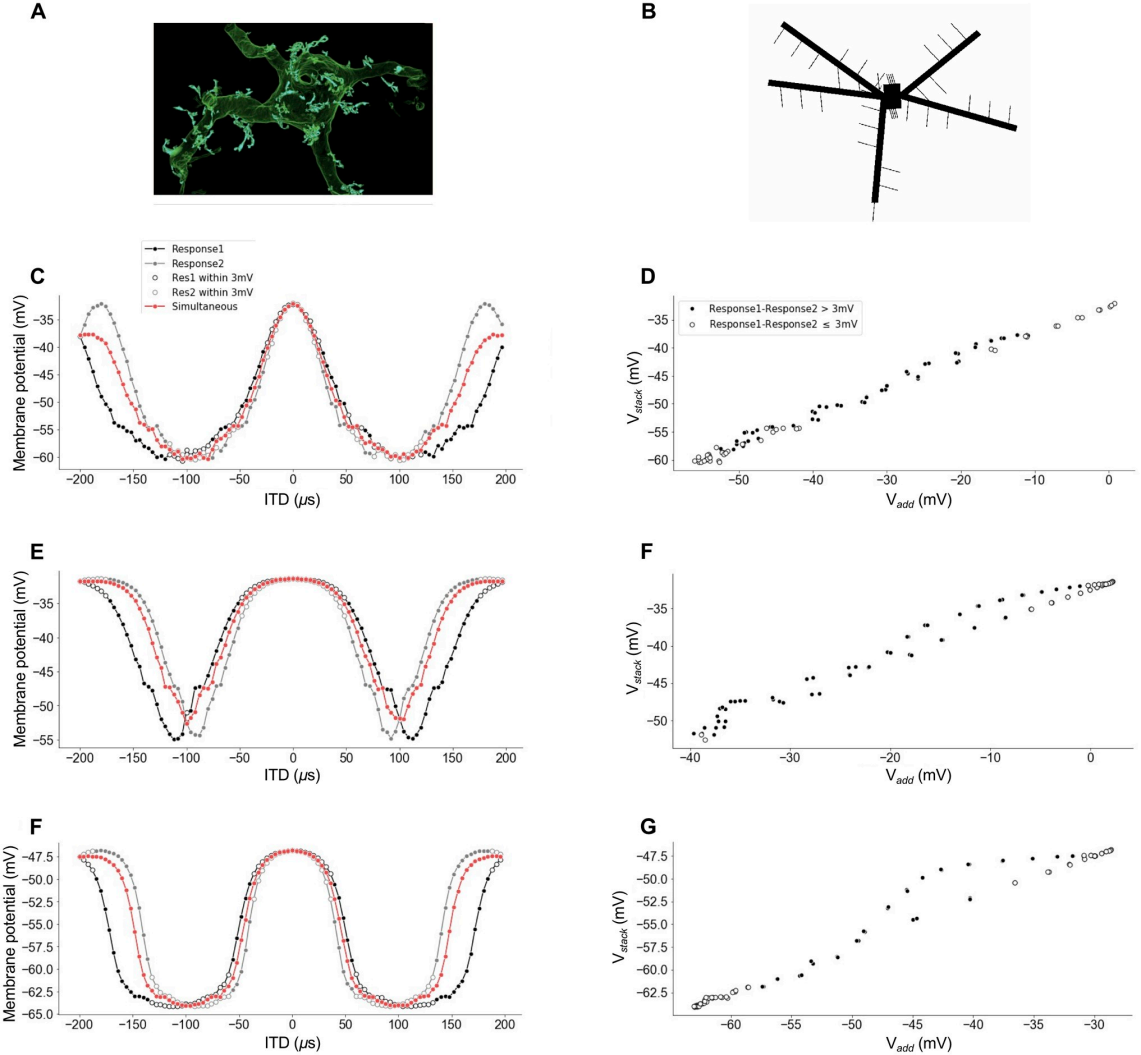
Modeling frequency integration in ICx using compartmental models. (A) Morphology of an ICx neuron, from [26]. (B) Compartmental model built using NEURON. Responses to independent and simultaneous stimuli (C,E,G) as a function of ITD, and the corresponding relationships between *V*_*add*_ and *V*_*stack*_ (D,F,H) for model configurations that produce linear combination created with AMPA synapses on the dendrites (C,D), quadratic combination created with AMPA synapses on the spines (E,F), and sigmoidal combination created with NMDA synapses on the spines (G,H).

Simulations using compartmental models showed that changes to the morphology, membrane parameters, and synapse types of an ICx neuron model with a passive membrane can result in inputs that combine linearly or nonlinearly. A compartmental model was built using the NEURON simulator, based on morphological (Fig 7A) and physiological properties of ICx neurons [26].

We found four key factors that influenced whether dendritic integration was linear or nonlinear: the type of synapse used (AMPA or NMDA), the diameter of the dendrite or spine which the input is on, the strength of the synaptic input, and the passive membrane conductance. Three of the factors (diameter, input strength, and conductance) have a similar effect of altering the saturation of the model neuron and thus can be grouped together.

We found that altering conductance, diameter, and synaptic weight could lead to nonlinear combination by increasing the saturation in the response. Models with larger conductance values (Fig 7C and 7D) combine more linearly than models with smaller conductances (Fig 7E–7H) [46]. Placing inputs on dendrites, with a diameter of 5 microns, leads to linear combination (Fig 7C and 7D); whereas, placing inputs on spines, with much smaller diameters (0.1–0.4 microns), leads to nonlinear combination (Fig 7E–7H) [30]. Stronger input weight and higher firing rates also lead to nonlinear combination (Fig 7E–7H) [46]. Furthermore, the nonlinearities observed in our model took either quadratic or sigmoidal forms and were consistent with the experimentally observed data. For synapse type, we found that AMPA synapses could lead to either linear or nonlinear combination (linear for lower saturation and nonlinear for higher saturation). NMDA synapses also lead to both linear and nonlinear combination, but nonlinear combination was observed for lower levels of saturation than it was using AMPA synapses. These results show that the experimentally observed nonlinear responses can be consistent with the integration of synaptic input in a neuron with a passive membrane.

We observed different types of combination rules for the main peaks and the side peaks of ITD curves. AMPA synaptic input consistently combined linearly on the main peaks of ITD curves and could be linear or nonlinear on the side peaks of ITD curves. NMDA synaptic input combined linear on the main peak except for higher saturation cases in which the main peak combination was sigmoidal or quadratic. The model showed that the difference in combination rule on the main and side peaks can be attributed to the fact that the inputs from the two frequency channels were matched in strength at the main peak and were mismatched at the side peaks (Fig 7). Thus, the model showed that the combination rule was approximately linear when the two inputs were matched in strength and nonlinear when the two inputs were mismatched in strength (Fig 7). We confirmed this observation by altering the frequencies of input to the model. This showed that alterations of input frequency by as narrow of margins as 0.5–1 kHz could make vast differences in the shape of the side-peak nonlinearities; however, changing the input frequencies had minimal impact on combination along the main peaks of ITD curves because altering input frequency does not alter the alignment of the main peaks of the two frequencies.

We also investigated how input clustering influenced combination but found no significant differences in the combination of inputs with different types of clustering. We found no significant difference between models where two dendrites had exclusively synapses from ICcl inputs that preferred one frequency (4.5 kHz) and two other dendrites had exclusively synapses from ICcl inputs that preferred a second frequency (5.5 kHz), and models where each dendrite had the same number of synapses that preferred 4.5 kHz as synapses that preferred 5.5 kHz. Synapses were placed both near each other (10 microns apart) and distant from each other (80 microns apart). Furthermore, we found no difference between models where spines had clustered preferred frequencies (spines had only one synapse frequency) and models where spines had un-clustered preferred frequencies (spines had synapses from both preferred frequencies).

**Fig 8:**
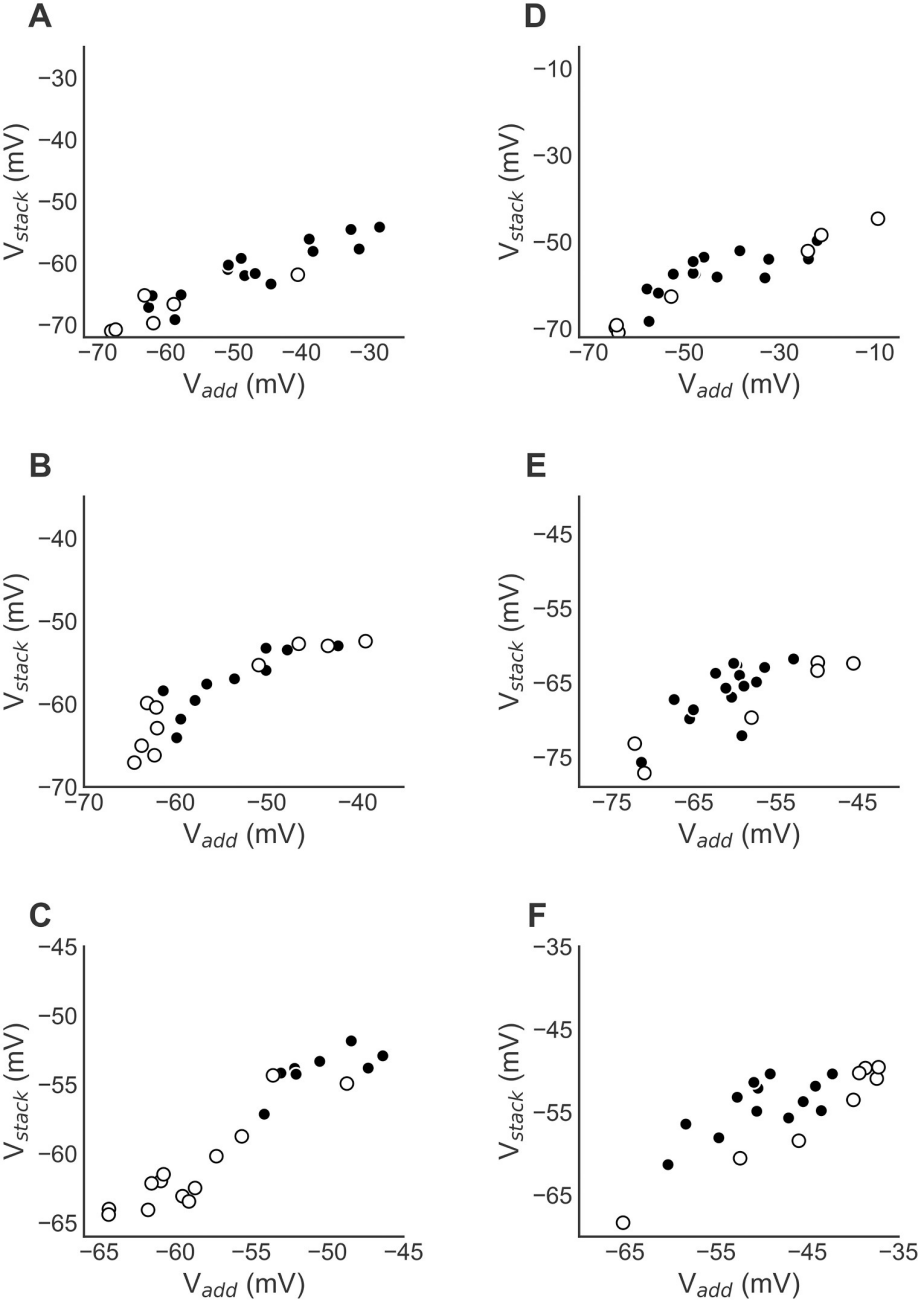
Testing compartmental model prediction. (A–C) Relationships between *V*_*add*_ and *V*_*stack*_ for the examples in Fig 3, which do not match the model predictions. Points where the responses to individual tones are matched (less than 3 mV apart) are shown in white. Modeling predicts that the relationship between *V*_*add*_ and *V*_*stack*_ should be linear for white points. (D–F). Examples where the relationship between *V*_*add*_ and *V*_*stack*_ is consistent with the model prediction.

The compartmental models led to the prediction that side peaks of ITD curves are more likely to combine nonlinearly than the main peaks. In particular, the compartmental model showed that the relationship between *V*_*add*_ and *V*_*stack*_ was linear when the responses to the individual tones at a particular ITD were similar (less than 3 mV apart; white dots in Fig 7D, 7F and 7H) and nonlinear when the responses to the individual tones at a particular ITD were not similar (greater than 3 mV apart; black dots in Fig 7D, 7F and 7H). The responses to the individual tones tend to be similar at the main peaks and different at the side peaks of the ITD curves. We tested whether this prediction is consistent with the physiological data but the results were inconclusive (Fig 8). The data shown in Fig 8 analyze the relationship between *V*_*add*_ and *V*_*stack*_ as seen in Fig 3B, 3D and 3F, but now with the data color coded to indicate where responses to individual tones are less than or greater than 3 mV apart using white and black dots, respectively. Modeling predicts that the relationship between *V*_*add*_ and *V*_*stack*_ should be linear for white points. Example neurons showed responses that were inconsistent with the prediction, where the combination rule was either linear (Fig 8A) or nonlinear (Figs 8B and 7C) for matched and mismatched responses to the individual tones. By contrast, other neurons had responses that were consistent with the model prediction, where the combination rule was linear for matched responses to the individual tones and nonlinear for mismatched responses to the individual tones (Fig 8D, 8E and 8F). Additional mechanisms should also be explored to explain the origin of nonlinear combination rules in ICx neurons.

The model results show that linear and nonlinear dendritic integration may occur in different ICx neurons through variation of passive membrane properties and synapse type within the ranges of experimentally observed values.

## Discussion

We showed the existence of ICx neurons with frequency integration responses mediated by linear and nonlinear integration of subthreshold input responses to sound. This demonstrates the presence of different functional types of neurons in ICx, based on cellular properties additional to the known spiking nonlinearity. Our two-layer model of ICx neurons showed that there are many combinations of ICcl-ICx connectivity and ICx cellular processing of inputs that can produce experimentally observed physiological responses. Furthermore, compartmental models showed there are multiple biophysical mechanisms that can produce the observed physiological responses based on morphology, membrane properties, and synapse types. These results show that processing of inputs at the site where the auditory map of space emerges is flexible, which may facilitate robust assembly of and adaptive changes in a circuit that supports learning.

The emergence of the auditory space map in ICx depends on the integration of information from multiple pathways in the sound localization system [7,8]. It has been shown that ICx neurons have nonlinear responses to combinations of ITD and ILD in both subthreshold membrane potentials and spiking responses [27]. Linear and nonlinear frequency convergence have been observed in ICx spiking responses [14,28]. However, frequency convergence in subthreshold membrane potential responses has been shown to be consistent with predictions of the linear cross-correlation model [18]. Furthermore, modeling suggested that linear across-frequency integration of subthreshold membrane potential inputs followed by a spiking nonlinearity could explain observed ICx spiking responses and would be beneficial for representing the locations of multiple simultaneous sounds [23]. Here, we used *in vivo* intracellular responses to tones and tone combinations to test these mechanisms. We found the presence of one type of responses consistent with the hypothesized linear-nonlinear point neuron model and a second type that required a subthreshold nonlinearity to describe frequency convergence in ICx. This demonstrates that rather than a stereotypical point-neuron model, a two-layer model with subunit nonlinearities is required to describe the responses of some ICx neurons.

Our modeling suggests that there is a high level of flexibility in the assembly of the ICcl-to-ICx circuit. There are multiple possibilities for both the connectivity between ICcl and ICx neurons and also for the form of the subunit nonlinearities. The model, however, required that there is frequency integration in the nonlinear subunits of ICx neurons showing nonlinear frequency convergence in the soma. This requirement is based on the assumption that the only substrate for nonlinear frequency integration prior to the recording site at the ICx neurons’ soma is the dendritic subunit nonlinearity. However, the model shows that diverse ranges of frequency convergence are possible, and therefore the learning rules used to establish the auditory space map do not need to result in a unique circuit configuration. Moreover, the two-layer model may be realized using different morphological and biophysical bases. The large number of possible circuit solutions may increase fault-tolerance and the rate of learning by increasing the chance that developmental changes lead to a circuit configuration that establishes a map of auditory space in ICx.

The two-layer model has been successful in describing synaptic integration properties of multiple types of neurons. Functionally, previous work showed that dendritic nonlinearities improve the capacity of a neuron to store information [34,47] and perform computations on its input [48–50]. Here we show that subunit nonlinearities allow neurons to retain the flexibility of rewiring to learn new sensory-motor transformations. Specifically, a two-layer model with sigmoidal subunit nonlinearities would allow subunits to retain weak inputs that do not contribute to spiking responses on their own and are masked in the presence of additional coordinated strong inputs that push the response into the saturating point of the nonlinearity, thus effectively remaining silent. Such silent input could increase the ability to flexibly rewire the ICcl-ICx connectivity to adaptively shift the auditory space map. This is a possible function of the large toric spines found in some ICx neurons. In addition to providing flexibility in rewiring, a sigmoidal subunit nonlinearity would allow for flexibility in the processing of sounds of different bandwidths. The nonlinearity would create consistent responses to sounds of different bandwidths by making SPS in weak narrowband inputs similar to SPS in stronger broadband inputs.

The results showed that the type of neuron commonly used in artificial neural networks, i.e., point neurons having linear integration of inputs followed by a static nonlinearity, is present in the barn owl’s auditory system at a site of learning. ICx is the site where associations between auditory cues and directions in space is learned, corresponding to a modification of the connectivity between ICcl and ICx neurons [51]. Learning new relationships between ITD and space is also associated with an increase in the relative number of NMDA-mediated synaptic inputs [52], which may be a basis for changing the subunit nonlinearity in the two-layer model. This is supported by the compartmental model showing that NMDA-mediated inputs lead to nonlinear combination. Nonlinearities implemented as rectified linear functions (ReLU) are preferred over sigmoid functions in deep neural networks because the saturating portion of the sigmoid nonlinearity slows down the gradient-based learning rules used to train deep neural networks. While the ReLU nonlinearity may be preferred for some learning-based considerations, the sigmoid nonlinearity has an expansive part that is important for computation. In the barn owl, it has been shown that the expansive mapping from membrane potentials to spikes is important for resolving spatial ambiguity in ITD tuning of ICx neurons [18,24,27]. There are several unknowns that prevent a direct generalization of our results to artificial neural networks. First, it is not known whether ICx neurons with linear and nonlinear subthreshold frequency integration play different roles in learning. Second, learning may rely on creating particular clusters of inputs in dendritic subunits [25,53], and not merely the adjustment of the synaptic weights of ICcl neurons that would be predicted by a typical artificial neural network.

Our results are limited in the ability to identify the form of the subunit nonlinearities in ICx. We compared membrane potential responses of ICx neurons to tones and tone-combinations to identify nonlinearity in subthreshold responses. Although we showed that the relationship between *V*_*add*_ and *V*_*stack*_ was nonlinear in a majority of neurons, this analysis does not uniquely determine the form of the nonlinear function that is applied to the ICcl inputs at the point of frequency convergence. However, the compartmental model showed that there are specific properties that may influence the linearity of combination. Specifically, the model showed that passive membrane properties that lead to higher saturation and higher densities of NMDA synapses may lead to nonlinear combination in ICx. To determine the form of the subunit nonlinearities, experiments must be performed that control or measure simultaneously the inputs and outputs of local regions of an ICx neuron. Moreover, because the data were collected from *in vivo* responses to sound, it is not possible to directly determine that the source of the subunit nonlinearity was subthreshold processing in ICx, rather than processing at an earlier stage of the pathway. However, the extensive knowledge of ITD tuning at previous stages of the pathway suggests that the source of the subunit nonlinearity is subthreshold processing in ICx. First, it has been shown that ITD tuning curves of neurons early in the sound localization pathway are similar for tonal and broadband noise stimuli, when compared for ITDs within the physiological range [22], which suggests that the responses of ICcl neurons to tones and tone combinations should be similar and thus not the source of nonlinearity in frequency convergence. Additionally, the network model showed that a wide range of nonlinear functions was possible at the first stage of the two-layer model of ICx neurons, but most of these nonlinear functions were suppressive, which argues against these nonlinearities corresponding to spiking nonlinearity of a second layer of neurons in ICcl. This is because the data used to constrain the model were collected for sounds presented at levels below saturation for most ICcl and ICx neurons [54,55]. Therefore, spiking nonlinearities of ICcl neurons may not be involved. Finally, although the majority of neurons in our sample had a nonlinear relationship between *V*_*add*_ and *V*_*stack*_ (16/20), we cannot state that this ratio will generalize to the larger ICx population. It is also possible that the use of ketamine anesthesia in the experiments influenced the linearity of the response, but it has been shown that spectral tuning is robust to the presence of anesthesia in midbrain neurons [56]. Further experiments are required to confirm the presence of, and to identify the exact form of, nonlinear subthreshold processing in ICx.

In sum, these results show a diversity of integration properties in neurons at a vital site of the sound localization pathway where synthesis of unambiguous topographic spatial tuning and experience-dependent learning take place. This work leads to the prediction of a varied relationship between connectivity and local subthreshold processing that may result in diverse and flexible functional roles across cells types in the auditory space map.

## Materials and methods

### Ethics statement

The past animal protocol for acquisition of data used in this study followed the National Institutes of Health Guide for the Care and Use of Laboratory Animals and was approved by the Laboratory Animal Care and Use Committee of the California Institute of Technology.

### Data collection

Intracellular recordings used in this study consisted of a previously reported dataset [18]. Briefly, data were obtained from 14 adult barn owls of both sexes. Owls were anesthetized by intramuscular injection of ketamine hydrochloride (25 mg/kg Ketaset; Phoenix Pharmaceutical, Belmont, CA) and diazepam (1.3 mg/kg; Steris Laboratories, Phoenix, AZ). An adequate level of anesthesia was maintained with supplemental injections of ketamine.

All experiments were performed in a double-walled sound-attenuating chamber. Acoustic stimuli were delivered by an earphone-assembly consisting of a Knowles ED-1914 receiver as a sound source, a Knowles (Electronics, Franklin Park, IL) BF-1743 damped coupling assembly for smoothing the frequency response of the receiver, and a calibrated Knowles 1939 microphone for monitoring sound pressure levels in the ear canal. Calibration data collected at the start of recording sessions contained amplitudes and phase of acoustic signals of both earphones across frequency. Irregularities in the frequency response of each earphone were automatically smoothed from 2 kHz to 12 kHz. Sounds were digitally synthesized and delivered by a stereo analog interface (DD1, Tucker-Davis Technologies, Gainesville, FL). Tonal stimuli 100 ms in duration, 5 ms linear rise/fall time, were presented once per second. ITD was varied in steps of 30 μs. Sharp borosilicate glass electrodes filled with 2 M potassium acetate and 4% neurobiotin were used for intracellular recording of ICx neurons. Analog signals were amplified (Axoclamp 2A, Axon Instruments, Foster City, CA) and stored in a computer. These unique data files were used for the current study. ITD and frequency tuning curves were computed for spiking and post-synaptic potentials (PSPs) responses using custom MATLAB (Mathworks, Natick, MA) software. To assess sound-evoked PSPs, the median membrane potential of the 50 ms of the response to sound starting 10 ms after onset was computed across stimulus trials and averaged over 3–5 repetitions of the same stimulus [24,27]. Using the median to summarize the membrane potential limits the influence of action potentials on the membrane potential response [45]. To measure spiking responses, spikes were detected by the data acquisition equipment and their timing was recorded in the data files. Spike counts were averaged across stimulus trials for each stimulus condition.

### Two-layer model of ICx neurons

We used a two-layer model of ICx neurons to investigate the role of nonlinear dendritic processing in producing ICx responses [33]. This model assumes that synaptic integration initially occurs within independent dendritic subunits and that subunit outputs combine linearly at the soma to drive a nonlinear spiking response (Fig 1). The two-layer model is a functional model where the dendritic subunit activity may correspond to various parts of the dendritic tree and may reflect both electrical and biochemical activity [33,57].

The two-layer model is a cascade of linear-nonlinear units that predicts the firing rate response of an ICx neuron from the responses of its input neurons. The inputs to ICx are from neurons in the lateral shell of the central nucleus of the inferior colliculus (ICcl) [13,32,51,58,59]. In the first layer of the ICx model, ICcl inputs are processed within *N* dendritic subunits that have a linear-nonlinear functional form. The response of the *j*^*th*^ dendritic subunit is given by

$$d_{j}=g_{i}\left(\sum_{i=1}^{M}w_{ij}r_{ICcl,i}\right),$$

where *r*_*iCcl*,*i*_ is the firing rate response of the *i*^*th*^ ICcl neuron, *w*_*ij*_ is a connection weight, *M* is the number of ICcl neurons, and *g*_*i*_ is a nonlinear function. In the second layer of the model, the dendritic subunit responses are added and passed through a spiking nonlinearity to produce the response of the ICx neuron. The firing rate response of the ICx neuron is given by

$$r=f(\sum_{j=1}^{N}d_{j})$$

where *f* is the spiking nonlinearity.

### Fitting the ICx spiking nonlinearity

We compared rectified-linear and sigmoid models of the spiking nonlinearity of ICx neurons responses by fitting these models to *in-vivo* intracellularly recorded responses to sounds. The spiking nonlinearity was defined as the mapping from the trial-averaged median membrane potential to the trial-averaged spike count. Data used in the analysis were collected using broadband noise stimuli at a fixed stimulus level that was 20–30 dB above threshold for multiple ITD and ILD values, and tones at multiple stimulus frequencies. It has previously been shown that the mapping from the membrane potential to the spike count does not vary significantly for different stimuli [60], and therefore responses were pooled over stimuli to fit the spiking nonlinearity.

The spiking nonlinearity models (rectified-linear and sigmoid) tested were implemented as follows. The rectified-linear spiking nonlinearity was given by

$$f_{ReLU}(V)=\left\{\begin{array}{c} 0;V\leq -\frac{a_{0}}{a_{1}}\\ a_{0}{+a}_{1}V;-\frac{a_{0}}{a_{1}}<V \end{array}\right.,$$

where *a*_1_ is the slope of the linear part of the function and $-\frac{a_{0}}{a_{1}}$ is the threshold.

While the rectified-linear model is linear above threshold, the rectification creates a nonlinearity that could account for differences in subthreshold and spiking responses of ICx neurons [23].

The sigmoid spiking nonlinearity was given by

fSV=0;V<αc01-e-c1V-c2;α≤V.

Model parameters *a*_0_, *a*_1_ and *c*_0_, *c*_1_, *c*_*2*_, *α* were jointly fit by minimizing the mean squared error.

We used two complementary methods to compare model fits for each neuron. Model fits were compared using the adjusted *R*^2^ and the leave-one-out cross validation (LOOCV) mean square error (MSE). Using these metrics helps to prevent overfitting noise in the data. The adjusted *R*^2^ modifies Pearson’s *R*^2^ to penalize models with more parameters, and is given by

$${\text{adjusted}\;R}^{2}=1-\frac{RSS/(n-p-1)}{TSS(n-1)}$$

where *RSS* is the residual sum of squares, *TSS* is the total sum of squares, *n* is the number of data points and *p* is the number of model parameters. The LOOCV MSE measure is obtained by fitting the model on all but one of the data points, then computing the MSE between the model prediction and the value of the held-out data point. The LOOCV MSE is the average over all *n* data points of this prediction error. We report that the data for a neuron was best fit by either the rectified-linear or sigmoid models if the adjusted *R*^2^ was larger and the LOOCV MSE was smaller for that model.

### Testing nonlinear subthreshold frequency integration in ICx

We tested nonlinear frequency integration in ICx subthreshold responses using *in vivo* intracellularly measured ITD tuning curves for tones and combinations of tones. Two or three tones were presented individually and in combination at a stimulus level that was the same for the individual tones and the tone-combination. The stimulus level was 20–30 dB above threshold. The response to the tone combination (*V*_*stack*_ was compared to a linear combination of the responses to the individual tones (*V*_*add*_) to test for nonlinear frequency integration in ICx subthreshold responses. The linear combination *V*_*add*_ is the sum of the responses to individual tones relative to the minimum membrane potential recorded during stimulation.

A linear fit between *V*_*add*_ and *V*_*stack*_ was compared to quadratic and sigmoidal fits to test for nonlinearity.

The sigmoidal function fit to the data was the sum of the standard sigmoid function and a linear term [33]:

$${\hat{V}}_{stack}=b_{0}+b_{1}V_{add}+\frac{b_{2}}{1-e^{-b_{3}(V_{add}-b_{4})}}.$$

The parameters of each model were jointly fit by minimizing the mean squared error. The best fit was determined using the adjusted *R*^2^ and the LOOCV MSE. We classified a neuron as being best fit by the linear, quadratic, or sigmoidal models if the adjusted *R*^2^ values was largest and the LOOCV MSE was smallest for one of the three models.

### Possible ICx subunit models

We used known properties of intracellularly recorded ITD tuning curves of ICx neurons [24] and ITD tuning in ICcl [61] to determine possible forms for dendritic subunit models. The dendritic subunit model has a linear integration step followed by a nonlinear response:

$$d_{j}=g_{i}(\sum_{i=1}^{M}w_{ij}r_{ICcl,i}).$$

The linear integration of ICcl responses $\sum_{i=1}^{M}w_{ij}r_{ICcl,i}$ determines the bandwidth of frequency integration that occurs in the subunit. The nonlinear response *g*_*i*_ determines whether the subunit has a suppressive or expansive effect on its inputs. The linear and nonlinear components of the subunit model together determine the relationship between frequency tuning bandwidth and ITD tuning shape in the subthreshold membrane potential of the ICx neuron.

The firing rate responses of ICcl neurons were simulated with a model that describes responses to tones and broadband noise. Neurons in ICcl have approximately sinusoidal ITD tuning for tone and broadband noise stimuli [61]. The ITD tuning curves are narrower than a sine wave, with a half-width that depends on the best frequency of the neuron [61]. We modeled these responses as

$$r_{ICcl,i}=(e^{b_{i}\left(\text{cos}\left(2\pi {bf}_{i}\left(ITD-{ITD}_{i}\right)\right)\right)}-e^{-b_{i}})/(e^{b_{i}}-e^{-b_{i}})$$

where *bf*_*i*_ is the best frequency, *ITD*_*i*_ is the best ITD, and *b*_*i*_ determines the width of the ITD tuning curve. The half-peak width, as a percentage of the period of the best frequency, shown in Fig 3B of [61] was well-described as a quadratic function of *b*_*i*_. Therefore, the parameter *b*_*i*_ was set as the solution to the quadratic equation

$$b_{i}=\frac{(17-\sqrt{17^{2}-4\times 2.8\left(24.12-2.44{bf}_{i}\right)}}{2\times 2.8}$$

to produce the relationship between best frequency and ITD tuning curve half-width shown in Fig 3B of [61]. The ICcl population model included neurons with best frequencies between 0.5 and 10 kHz and best interaural time differences between -250 and 250 μs [62].

We tested ICx subunit models with different frequency integration bandwidths and nonlinearity shapes to determine which models produced subthreshold ITD tuning curves that were consistent with experimentally measured intracellular responses in ICx.

The frequency integration bandwidth of the ICx model neuron was controlled by the connection weights *w*_*ij*_ in the linear stage of the model and the range of subunit center frequencies. The connection weights were a Gaussian-shaped function of the difference between the center frequency of the ICx subunit and the best frequencies of the ICcl neurons:

$$w_{ij}=exp(-\frac{1}{2}{\left(\frac{{bf}_{ICcl,i}-{cf}_{ICx,j}}{\sigma }\right)}^{2}).$$

The value of *σ* determines the frequency integration bandwidth and was varied between 0.1 to 4 kHz. The subunit center frequencies *c*_*fICx*,*j*_ were selected from different ranges (0, 1.25, and 2.5 kHz) centered at 5 kHz. In each simulation, the model contained 10 dendritic subunits. A value of 5 kHz was selected for the center of the range because the experimentally measured ITD tuning curves are from neurons with preferred ITDs near zero [24] and these neurons prefer higher frequencies [63].

We tested subunit nonlinearities that ranged from suppressive to expansive functions of their input. The subunit nonlinearity was a power function

$$g_{i}(x)=x^{p}$$

where the power *p* ranged from 0.1 to 10. The subunit nonlinearity is suppressive for *p* < 1 and expansive for *p* > 1.

We used two properties of the subthreshold ITD tuning curves to constrain the model: the half-peak width (HW) and the side-peak suppression (SPS). The subunit models were evaluated by computing the SPS and HW from ITD tuning curves in the model subthreshold potential $V=\sum_{j=1}^{N}d_{j}$ and comparing the values to the experimentally measured distributions. Population distributions of these parameters have been described previously [24]. The mean and standard deviation of the SPS are 23.18% and 12.27%, respectively, and the mean and standard deviation of the HW are 75.17 *μs* and 17.12 *μs*, respectively [24]. These means and standard deviations were used to compute z-scores of SPS and HW values produced by model neurons. Large absolute values of the z-scores would indicate that the model is inconsistent with the experimental measurements.

### Compartmental model of ICx neurons

The two-layer model suggests that ICx neurons combine inputs across frequency channels using both linear and nonlinear combination rules. In order to investigate potential mechanisms underlying different input-combination properties in the barn owl’s ICx neurons, a compartmental model of ICx neurons was designed using the NEURON simulator [64]. This biophysical compartmental model allowed a more mechanistic testing than the two-layer model by considering how cellular morphology, membrane properties, and synapse types influence whether inputs are integrated linearly or nonlinearly.

We used morphological data from a detailed reconstruction of an ICx neuron to constrain the diameter and length of the soma and dendrites of model ICx neurons [26]. Assuming that spines could be modeled as cylinders, we used Sanculi et. al.’s (2020) measurements of spines’ volumes and surface areas to generate realistic ranges of the diameter and length of the model’s spines that cover the ranges observed for both toric and normal spines. Using cylinder formulas (*Volume* = *πr*^2^*h* and *Surface area* = 2 *πrh* + 2 *πr*^2^ where *r* and *h* are the radius and height of the cylinder, respectively) and the measured upper and lower bounds of volume and surface area, we solved for ranges of the radius and height of the cylindrical spines. This resulted in the ranges: diameter (0.1–0.4 microns) and length (2–80 microns). The model was assigned mean spine diameters and lengths within these ranges, and individual spines varied randomly within an interval 10% above and below the mean. Furthermore, we avoided morphologies that took the minimum diameter and maximum length, and vice versa, in order to avoid unrealistic models [26]. The model dendrites varied in length from 50 microns to 300 microns and in diameter from 4 microns to 20 microns; and the soma varied in diameter and length both from 10 to 30 microns. The soma and dendrite ranges are based on visual approximations of the length and diameter of the soma and dendrites of a reconstructed ICx neuron [26].

We varied the passive membrane conductance of the spines, dendrites and soma. We allowed the passive membrane conductance to be uniform throughout the cell, or to vary between the soma, dendrites, and spines. We varied the conductance from 0.0001 to 0.01 *μ*S [26]. The model did not include voltage-dependent ionic channels that would produce action potentials.

The input to the compartmental model simulated the tone and tone combination stimuli used in the *in vivo* intracellular recordings described above. Model ICcl synaptic inputs were assigned one of two preferred frequencies that ranged between 2 and 8 kHz. The model was stimulated with two sound inputs playing independently and then simultaneously. The firing rates of model ICcl neurons in response to these sounds were governed by the following equation that outputs the firing rate of input spikes:

$$b_{i}=\frac{(17-\sqrt{17^{2}-4\times 2.8\left(24.12-2.44f_{{in}_{i}}\right)}}{2\times 2.8}$$

$$\begin{array}{cc} Firing\;Rate= & w_{1}\left(\frac{e^{b_{1}\left(\text{cos}\left(2\pi {bf}_{i}\left({ITD}_{pref}-{ITD}_{in}\right)\right)\right)}-e^{-b_{1}}}{e^{b_{1}}-e^{-b_{1}}}\right)e^{-.5\left({\left(\frac{{f_{pref}-f}_{{in}_{1}}}{w_{2}}\right)}^{2}\right)}\\ & +w_{2}\left(\frac{e^{b_{2}\left(\text{cos}\left(2\pi {bf}_{i}\left({ITD}_{pref}-{ITD}_{in}\right)\right)\right)}-e^{-b_{2}}}{e^{b_{2}}-e^{-b_{2}}}\right)e^{-.5\left({\left(\frac{{f_{pref}-f}_{{in}_{2}}}{w_{2}}\right)}^{2}\right)}+\frac{1}{{int}_{max}} \end{array}$$

where *w*_1_ and *w*_2_, which are weight parameters, were adjusted such that the minimum interval varied between ~3ms and ~20ms; $f_{{in}_{1}}$ and $f_{{in}_{2}}$ are the two input frequencies (varied between 2 and 8Khz); *ITD*_*pref*_ is the preferred interaural time difference of the given synapse; *ITD*_*in*_ is the interaural time difference of the sound input (both *ITD*_*pref*_ and *ITD*_*in*_ were kept near 0); and *int*_*max*_ sets a maximum interval between input spikes (this was set to 100 ms and implemented to avoid dividing by 0). This equation yields the mean firing rate of a Poisson spike train.

We implemented both NMDA and AMPA synapses on the ICx model neurons [65,66]. For the NMDA synapses, we utilized a NEURON mod file created by Gasparini et al. (2004) [67,68] that is defined by the equations:

$$g\left(V\right)=\frac{1}{1+\text{exp}\left(-0.062V\right)\left(\frac{C}{3.57}\right)}\cdot \left(Ron+Roff\right)\cdot 1\mu \Omega$$

$$R_{off}\prime =-\beta \cdot R_{off}$$

$$R_{on}\prime =\left\{\begin{array}{cc} \frac{R_{inf}-R_{on}}{R_{tau}} & if\;t=t_{spike}\\ \frac{-R_{on}}{R_{tau}} & if\;t\neq t_{spike} \end{array}\right\}$$

where *V* is membrane potential in mV, *C* is extracellular magnesium concentration in mM, *g* is the conductance in *μ*S, *R*_*tau*_ is the time constant of channel binding and is defined as $R_{tau}=\frac{1}{\alpha +\beta }$ where *α* is the binding rate (ms) and *β* is the unbinding rate (ms), and $R_{inf}=c\;\frac{\alpha }{\alpha +\beta }$ where *c* is a steady state constant for when all the channels are open. AMPA synapses were implemented using NEURON’s built in AMPA synapse denoted “ExpSyn.” The AMPA synaptic conductance following a spike at time *t*_*spike*_ is modeled using the equation:

$$g\left(t\right)=weight∙e^{\frac{-\left(t-t_{spike}\right)}{\tau }},t>t_{spike}$$

where *g* is the conductance in *μ*S, *t* is the time, *τ* = 4.5 *ms* is the time constant, and *weight* is a weight parameter.

Finally, we determined whether the relationship between the sum of independent inputs and the response of the simultaneous inputs was linear, quadratic, or sigmoidal, to compare with the experimental results. Furthermore, we evaluated how combination at the main peak of the ITD curves differed from combination on the side peaks.

## Supporting information

S1 Table Adjusted R^2^ values for model fits of the spiking nonlinearity. The adjusted R^2^ value measures the accuracy of the model fit with a penalty added based on the number of model parameters (see Materials and methods).
(PNG)

S2 Table Leave-one-out cross validation mean square error values for model fits of the spiking nonlinearity. The leave-one-out cross validation mean square error measures the accuracy of the model fit on data not used in the model fit, to avoid overfitting the data (see Materials and methods).
(PNG)

S3 Table Adjusted R^2^ values for model fits of the relationship between *V*_*add*_ and *V*_*stack*_. The adjusted R^2^ value measures the accuracy of the model fit with a penalty added based on the number of model parameters (see Materials and methods).
(PNG)

S4 Table Leave-one-out cross validation mean square error values for model fits of the relationship between *V*_*add*_ and *V*_*stack*_. The leave-one-out cross validation mean square error measures the accuracy of the model fit on data not used in the model fit, to avoid overfitting the data (see Materials and methods).
(PNG)
