# Supplementary figures and images for: Diverse processing underlying frequency integration in midbrain neurons of barn owls
Source: PLoS Comput Biol. 2021 Nov 11;17(11):e1009569. doi: 10.1371/journal.pcbi.1009569 (PMC8610287; doi:10.1371/journal.pcbi.1009569)

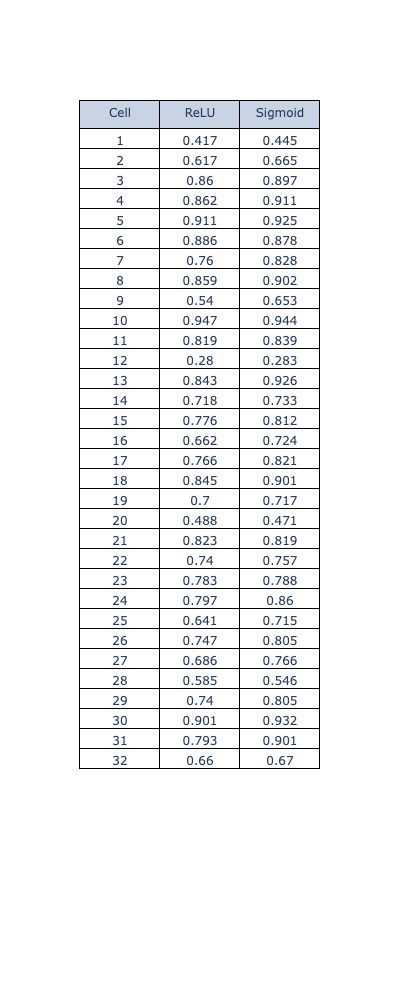

Supplement: S1 Table — The adjusted R2 value measures the accuracy of the model fit with a penalty added based on the number of model parameters (see Materials and methods). (PNG) [file pcbi.1009569.s001.png]

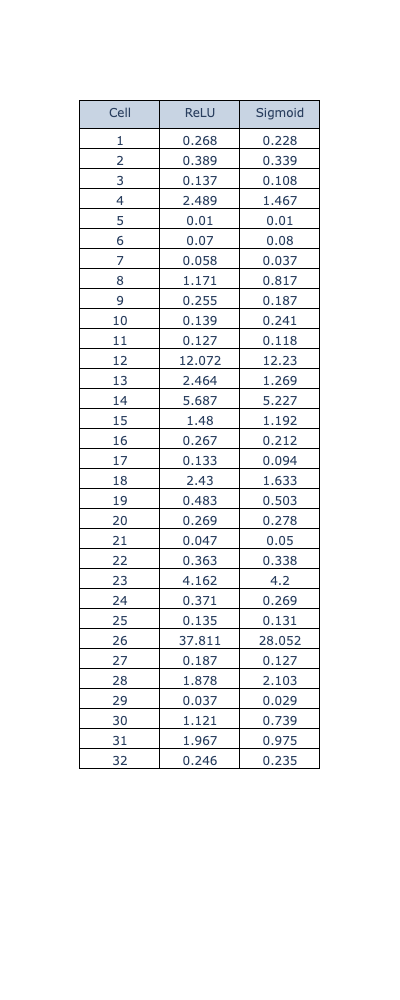

Supplement: S2 Table — The leave-one-out cross validation mean square error measures the accuracy of the model fit on data not used in the model fit, to avoid overfitting the data (see Materials and methods). (PNG) [file pcbi.1009569.s002.png]

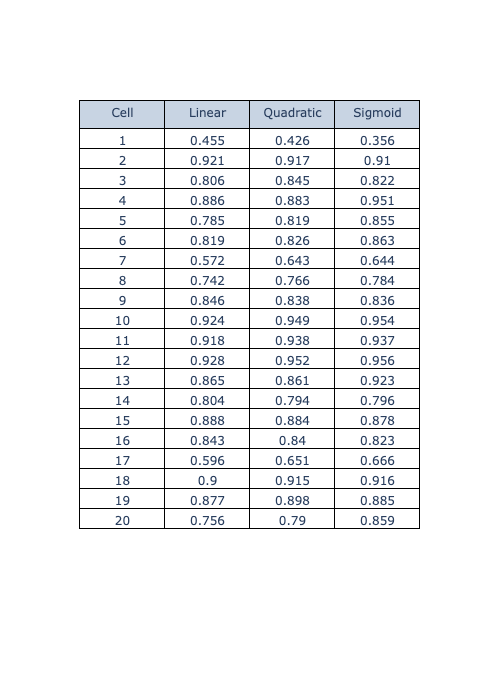

Supplement: S3 Table — The adjusted R2 value measures the accuracy of the model fit with a penalty added based on the number of model parameters (see Materials and methods). (PNG) [file pcbi.1009569.s003.png]

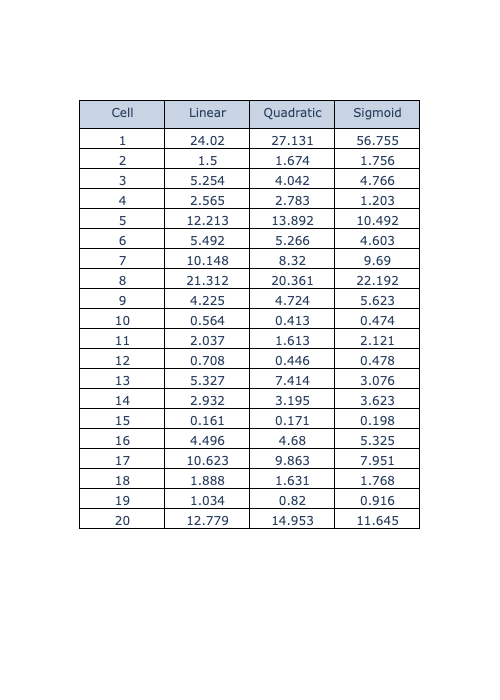

Supplement: S4 Table — The leave-one-out cross validation mean square error measures the accuracy of the model fit on data not used in the model fit, to avoid overfitting the data (see Materials and methods). (PNG) [file pcbi.1009569.s004.png]
